# Concomitant palonosetron ameliorates cisplatin-induced nephrotoxicity, nausea, and vomiting: a retrospective cohort study and pharmacovigilance analysis

**DOI:** 10.1186/s40780-022-00252-z

**Published:** 2022-08-01

**Authors:** Miho Takemura, Kenji Ikemura, Masayoshi Kondo, Fumihiro Yamane, Mikiko Ueda, Masahiro Okuda

**Affiliations:** 1 Department of Clinical Pharmacy Research and Education, Graduate School of Pharmaceutical Sciences, Osaka University, 1-6 Yamadaoka, Suita, Osaka, 565-0871 Japan; 2 Department of Pharmacy, Osaka University Hospital, 2-15 Yamadaoka, Suita, Osaka, 565-0871 Japan; 3 Department of Hospital Pharmacy, Graduate School of Pharmaceutical Sciences, Osaka University, 1-6 Yamadaoka, Suita, Osaka, 565-0871 Japan

**Keywords:** Cisplatin, Nephrotoxicity, Chemotherapy-induced nausea and vomiting, 5-hydroxytryptamine type 3 receptor antagonist, Palonosetron

## Abstract

**Background:**

Cisplatin (CDDP)-induced nephrotoxicity is the most important complication of CDDP treatment. 5-Hydroxytryptamine type 3 receptor antagonists (5-HT_3_RAs) are widely used to prevent chemotherapy-induced nausea and vomiting (CINV). However, in patients with the triple antiemetic (neurokinin-1 receptor antagonist, 5-HT_3_RA, and dexamethasone) therapy, the advantage of palonosetron in comparison with other 5-HT_3_RAs on CDDP-induced nephrotoxicity and CINV remains unclear. In the present study, we investigated the effect of palonosetron on CDDP-induced nephrotoxicity and CINV in patients with the triple antiemetic therapy by a retrospective cohort study and a pharmacovigilance analysis.

**Methods:**

We retrospectively analyzed the effect of 5-HT_3_RAs on the development of nephrotoxicity and CINV in 110 patients who received CDDP, fluorouracil, and triple antiemetic therapy for the treatment of esophageal cancer. Moreover, the effect of 5-HT_3_RAs on CDDP-induced nephrotoxicity was validated in patients with the triple antiemetic therapy using the Japanese Adverse Drug Event Report (JADER) database.

**Results:**

In a retrospective study, the incidence of nephrotoxicity (≥ grade 1) in patients receiving palonosetron (18%) was significantly lower than that in patients receiving ramosetron (another 5-HT_3_RA) (36%, *p* = 0.044). Moreover, severe nephrotoxicity ≥ grade 3 was observed in one patient treated with ramosetron, whereas hematological toxicity was comparable between the two groups (*p* = 0.553). Furthermore, the incidence rate of CINV within 120 h following CDDP administration in patients treated with palonosetron (18%) was significantly lower than that in patients receiving ramosetron (39%, *p* = 0.026). JADER database analyses revealed that the reporting odds ratio of palonosetron for CDDP-induced acute kidney injury was 0.282 (95% confidence interval: 0.169–0.472).

**Conclusions:**

The findings of the present study suggested a greater potential of palonosetron against CDDP-induced nephrotoxicity and CINV than other 5-HT_3_RAs in patients with the triple antiemetic therapy.

## Background

Cisplatin (CDDP) is a platinum-based drug that is widely used as first-line chemotherapy for various solid tumors, including lung, ovarian, bladder, testicular, head and neck, esophageal, gastric, and pancreatic cancers [1]. However, the use of CDDP is limited by occurrence of severe side effects in normal tissues, particularly nephrotoxicity. CDDP-induced nephrotoxicity occurs in approximately one-third of the patients receiving CDDP treatment, despite intensive prophylactic measures [2]. Therefore, co-administration of medicines with renal protective effects is crucial for the prevention of severe and irreversible damage to the kidney, and for the success of CDDP chemotherapy.

CDDP treatment has been classified as highly emetogenic chemotherapy [3]. The guidelines for antiemetic treatment recommend the use of triple antiemetic drugs (neurokinin-1 receptor antagonist, 5-hydroxytryptamine type 3 receptor antagonist (5-HT_3_RA), and dexamethasone) for cancer patients receiving highly emetogenic chemotherapy, including CDDP regimen [4–6]. The current guidelines recommend palonosetron as the preferred 5-HT_3_RA for preventing both acute and delayed chemotherapy-induced nausea and vomiting (CINV) in patients receiving both moderately and highly emetogenic chemotherapeutic regimens [4–6]. A previous retrospective study reported that palonosetron suppressed CDDP-induced increases in serum creatinine (Scr) and blood urea nitrogen (BUN) levels from clinical data treated with CDDP and 5-HT_3_RAs [7]. Furthermore, an analysis using the US Food and Drug Administration Adverse Event Reporting System and retrospective medical records revealed that first-generation 5-HT_3_RAs (ondansetron, granisetron, or ramosetron) significantly increased renal adverse events associated with CDDP as compared with a second-generation 5-HT_3_RA, palonosetron [8]. However, the advantage of palonosetron on CDDP-induced nephrotoxicity and CINV in comparison with other 5-HT_3_RAs remains unclear in patients with the triple antiemetic therapy.

In the present study, we retrospectively evaluated the effect of palonosetron on the development of nephrotoxicity and CINV in patients receiving CDDP, fluorouracil (5-FU), and triple antiemetic therapy by a retrospective cohort study and a pharmacovigilance analysis using the Japanese Adverse Drug Event Report (JADER) database.

## Methods

### Patients selection

Data of 122 patients hospitalized in Osaka University Hospital between January 2010 and December 2020, who received CDDP, 5-FU, and triple antiemetic therapy for the first time for the treatment of esophageal cancer and received triple antiemetic therapy, were extracted from the electronic medical records. Eligible patients received a continuous infusion of 5-FU (800 mg/m^2^) for 5 days, a 2-h intravenous infusion of CDDP (80 mg/m^2^), an oral aprepitant (125 mg on day 1 and 80 mg on days 2 and 3), an intravenous infusion of dexamethasone (6.6 mg on day 1 through 4), and an intravenous infusion of ramosetron (0.3 mg on day 1 through 4) or palonosetron (0.75 mg on day 1). Patients were excluded if they had missing data, baseline Scr > 1.3 mg/dL, BUN > 22 mg/dL, aspartate aminotransferase (AST) and alanine aminotransferase (ALT) > 100 IU/L, and hematological parameters grade ≥ 2 before chemotherapy, including white blood cell (WBC) count, platelet (PLT) count, absolute neutrophil count (ANC), or hemoglobin (Hb) level, defined as the Common Terminology Criteria for Adverse Events (CTCAE) version 5.0.

### Evaluation of side effects following chemotherapy

We investigated the effect of 5-HT_3_RAs on the maximum values of Scr and BUN within 14 days following CDDP administration, the duration when CDDP-induced nephrotoxicity is usually observed [2]. In addition, the severity of nephrotoxicity within 14 days following CDDP administration was evaluated in accordance with the criteria for acute kidney injury defined as the CTCAE version 4.0 [9]. Hematological toxicity that developed within 28 days after CDDP administration was defined as grade ≥ 3 for WBC, PLT, ANC, or Hb. The incidence rates of CINV during the acute (0–24 h), delayed (24–120 h), and overall phase (0–120 h) following CDDP administration were investigated. Diabetes mellitus was defined by continued treatment with hypoglycemic drugs and fasting plasma glucose ≥126 mg/dL. Cardiovascular disease was defined as angina or myocardial infarction. Urinary and infusion volumes were calculated as the cumulative amount for 3 and 5 days following CDDP administration, respectively. The primary endpoint was the incidence of grade ≥ 1 nephrotoxicity. The secondary endpoints included the severity of nephrotoxicity, CINV, and hematological toxicity following CDDP administration.

### Analyses on the effect of 5-HT_3_RAs on CDDP-associated acute kidney injury using the JADER database

Data on patient demographic information (DEMO), drug information (DRUG), adverse events (REAC), and primary disease (HIST) from April 2004 to September 2021 were obtained from the JADER database released by the PMDA (https://www.pmda.go.jp/). Data associated with CDDP and triple antiemetic therapy were extracted. Disease names were defined using the Medical Dictionary for Regulatory Activities (MedDRA/J) version 24.0. According to a previous report [10], the following six preferred term was used for searching CDDP-associated acute kidney injury: “acute kidney injury,” “renal impairment,” “renal failure,” “renal disorder,” “renal function test abnormal,” and “renal tubular disorder.” Effect of 5-HT_3_RAs on CDDP-associated acute kidney injury was evaluated using the reporting odds ratio (ROR). To calculate the ROR, CDDP-associated acute kidney injury and all other reported adverse events associated with CDDP were defined as “cases” and “non-cases,” respectively. The RORs were calculated from two-by-two contingency tables of counts with or without 5-HT_3_RA. RORs were expressed as point estimates with 95% confidence interval (CI).

### Statistical analyses

Statistical comparisons between two groups were performed using the Mann-Whitney U test and Fisher’s exact test for continuous and categorical variables, respectively. Statistical analyses were performed using the GraphPad Prism version 8.4.3 (GraphPad Software Inc., San Diego, CA). A two-tailed *p*-value < 0.05 was considered statistically significant, and the confidence level was set to 95%.

## Results

### Patients’ characteristics

After considering inclusion and exclusion criteria, 110 of 122 patients were enrolled in the present study. Patient characteristics are summarized in Table 1. Forty-four patients (40%) received ramosetron, and 66 patients (60%) received palonosetron as 5-HT_3_RA. There were no significant differences in characteristics of patients treated with ramosetron and palonosetron.

**Table 1:** Patients’ characteristics

	Ramosetron (*n* = 44)	Palonosetron (*n* = 66)	*p*-Value
Age (years)	71 [51–84]	71 [48–84]	0.744
Male	37 (84)	54 (82)	0.803
Body weight (kg)	52.7 [33.0–80.0]	53.1 [32.2–79.2]	0.502
CDDP dose (mg)	110 [75–140]	113 [71–140]	0.424
5-FU dose (mg)	1110 [918–1400]	1137 [770–1400]	0.357
Infusion volume (L)	2.0 [1.4–5.0]	2.7 [1.5–3.7]	0.544
Urine volume (L)	5.6 [3.1–10.7]	5.6 [3.7–9.6]	0.145
Baseline biological parameters			
AST (U/L)	20 [9–57]	21 [13–50]	0.501
ALT (U/L)	14 [6–68]	17 [6–64]	0.081
Scr (mg/dL)	0.84 [0.43–1.15]	0.78 [0.44–1.25]	0.408
BUN (mg/dL)	14 [7–22]	16 [6–22]	0.505
WBC (×10^9^/L)	5.37 [3.48–8.83]	5.33 [2.68–9.29]	0.998
PLT (×10^9^/L)	234 [127–609]	217 [103–398]	0.101
ANC (×10^9^/L)	3.17 [1.86–6.72]	3.31 [1.65–6.96]	0.674
Hb (g/dL)	12.8 [9.4–16.2]	12.9 [9.2–16.2]	0.807
Medical history			
Diabetes mellitus	6 (14)	6 (9)	0.538
Cardiovascular disease	2 (5)	5 (8)	0.700
Co-administrated drugs			
Diuretics	6 (14)	7 (11)	0.765
NSAIDs	11 (25)	23 (35)	0.300
PPIs	22 (50)	41 (62)	0.241
Magnesium oxide	15 (34)	27 (41)	0.550

Values are presented as median [range] or number (%). Statistical analyses were performed using Fisher’s exact test or the Mann-Whitney U test

*5-FU* fluorouracil, *ALT* alanine transaminase, *ANC* absolute neutrophil count, *AST* aspartate transaminase, *BUN* blood urea nitrogen, *CDDP* cisplatin, *Hb* hemoglobin, *NSAIDs* non-steroidal anti-inflammatory drugs, *PLT* platelet, *PPI* proton pump inhibitor, *Scr* serum creatinine, *WBC* white blood cell

### Nephrotoxicity, hematological toxicity, and CINV following administration of CDDP and 5-FU administration in patients receiving ramosetron and palonosetron

The number of patients with nephrotoxicity and hematological toxicity after CDDP and 5-FU administration in patients receiving ramosetron and palonosetron are shown in Table 2. The incidence of nephrotoxicity in patients receiving palonosetron (18%) was significantly lower than that in patients treated with ramosetron (36%, *p* = 0.044). Furthermore, grade ≥ 3 nephrotoxicity was observed in one patient treated with ramosetron. In contrast, there was no significant difference in the incidence of hematological toxicity between patients treated with ramosetron and palonosetron (*p* = 0.553).

**Table 2:** Number of patients with nephrotoxicity and hematological toxicity following CDDP and 5-FU administration

	Ramosetron (*n* = 44)	Palonosetron (*n* = 66)	*p*-Value
Nephrotoxicity (*n* = 28)	16 (36)	12 (18)	0.044
Grade 1	12 (27)	11 (17)	
Grade 2	3 (7)	1 (2)	
Grade 3	1 (2)	0 (0)	
Hematological toxicity (*n* = 45)	16 (36)	29 (44)	0.553
Anemia	1 (2)	1 (2)	
Leukopenia	16 (36)	29 (44)	
Thrombocytopenia	1 (2)	1 (2)	
Neutropenia	2 (5)	3 (5)	

Values are presented as number (%). Hematological toxicity that developed within 28 days after CDDP administration was defined as grade ≥ 3 of WBC, PLT, ANC, or Hb. Statistical analyses were performed using the Fisher’s exact test

**Fig. 1:**
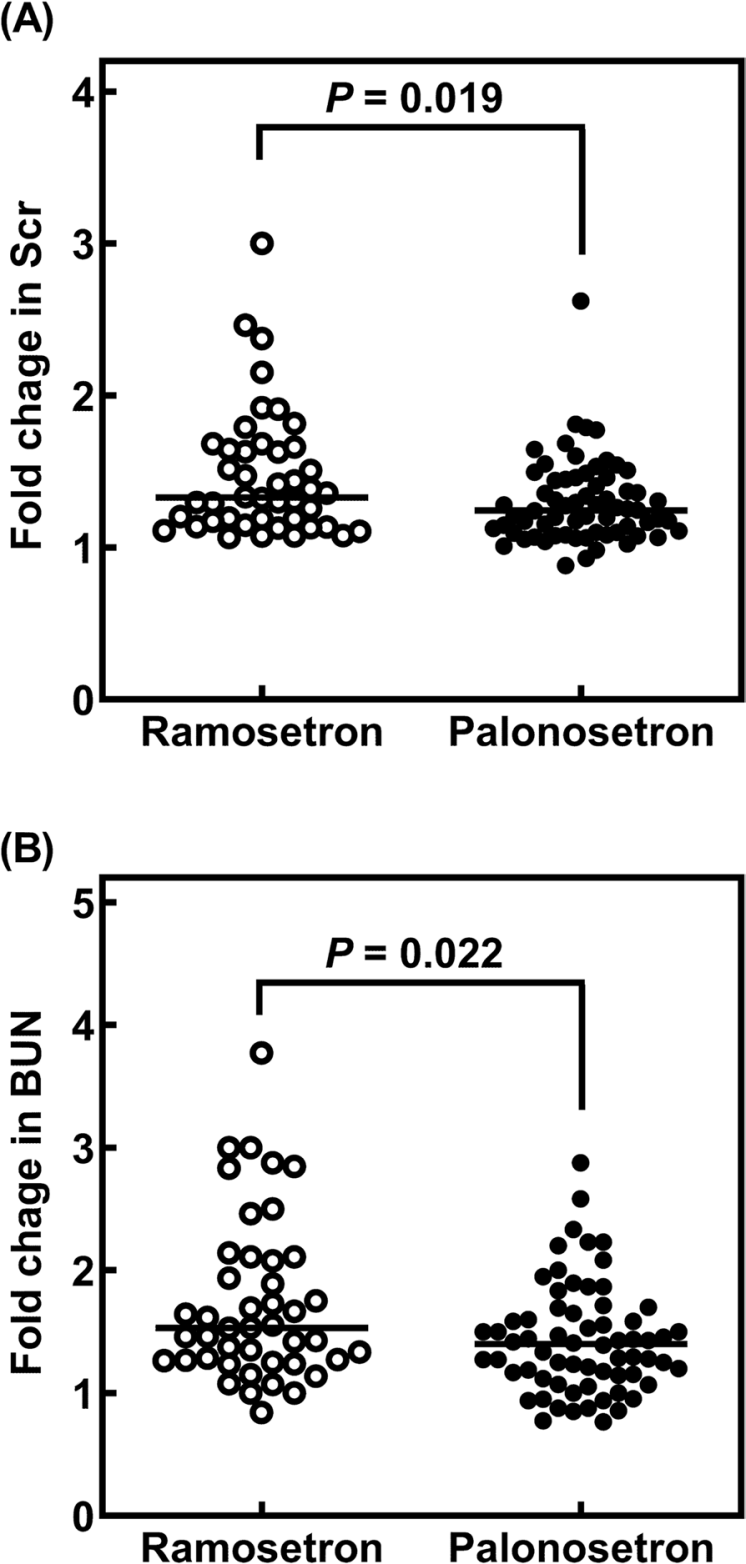
Comparison of the fold changes of (**A**) Scr and (**B**) BUN following CDDP, 5-FU, and triple antiemetic therapy in patients treated with ramosetron (*n* = 44) and palonosetron (*n* = 66)

Figure 1 shows the comparison of the fold changes in Scr and BUN following CDDP and 5-FU administration between patients receiving ramosetron and palonosetron. As shown in Fig. 1, the fold change of Scr and BUN in patients receiving palonosetron was significantly lower than that in patients treated with ramosetron (*p* = 0.019 and 0.022, respectively).

**Fig. 2:**
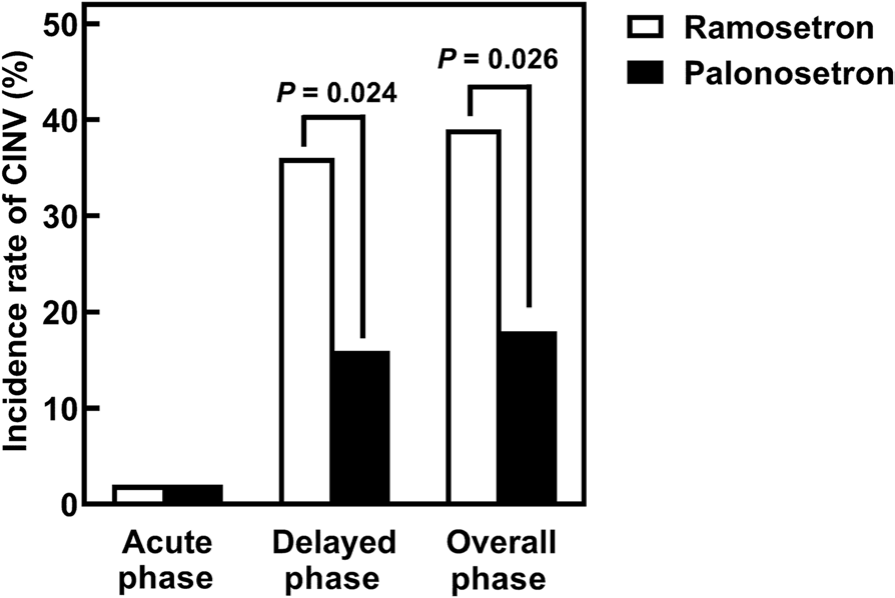
Comparison of incidence rates of chemotherapy-induced nausea and vomiting (CINV) following CDDP, 5-FU, and triple antiemetic therapy in patients treated with ramosetron (*n* = 44) and palonosetron (*n* = 66)

Figure 2 shows the comparison of incidence rates of CINV following CDDP administration between patients receiving ramosetron and palonosetron. During overall phase, the incidence rate of CINV in patients receiving palonosetron (18%) was significantly lower than in those receiving ramosetron (39%, *p* = 0.026). During the acute phase, nausea was observed in one patient (2%) each from ramosetron and palonosetron treatment groups. However, during the delayed phase, 16 patients (36%) treated with ramosetron and 11 patients (17%) with palonosetron developed nausea, with significantly low incidence rate of nausea in patients treated with palonosetron (*p* = 0.024). Furthermore, one patient (2%) each from ramosetron and palonosetron treatment groups had vomiting, however, severe vomiting of grade 2 was observed only in the patient treated with ramosetron.

### Analyses on the effect of 5-HT_3_RAs on CDDP-associated acute kidney injury in patients received with CDDP and triple antiemetic therapy using the JADER database

The 751,497 reports in the JADER database from April 2004 to September 2021 were analyzed. A total of 288 cases of CDDP-associated acute kidney injury were identified among a total of 635 cases received with CDDP and triple antiemetic therapy. The results of reporting ratio of CDDP-associated acute kidney injury and RORs with 95% CI in patients receiving CDDP and triple antiemetic therapy are summarized in Table 3. JADER database analyses revealed that the reporting odds ratio of palonosetron for CDDP-induced acute kidney injury was 0.282 (95% CI: 0.169–0.472), whereas there was absence of any significant signal for other 5-HT_3_RAs.

**Table 3:** Analyses on the effect of 5-HT_3_RAs on CDDP-associated acute kidney failure in patients received with CDDP and triple antiemetic therapy using the JADER database

	CDDP-associated acute kidney injury (%)		ROR (95% CI)
	Without drug	With drugs	
Granisetron	43/370 (12)	29/265 (11)	0.934 (0.567–1.540)
Ondansetron	69/609 (11)	3/26 (12)	1.021 (0.299–3.489)
Palonosetron	31/130 (24)	41/505 (8)	0.282 (0.169–0.472)
Ramosetron	72/599 (12)	0/36 (0)	0.100 (0.006–1.642)

Ratio of CDDP-associated acute kidney injury is presented as cases / (cases + non-cases) (%)

*5-HT*_*3*_*RA* 5-hydroxytryptamine type 3 receptor antagonist, *CI* confidence interval, *JADER* Japanese Adverse Drug Event Report, *ROR* reporting odds ratio

## Discussion

We identified the effect of palonosetron on CDDP-induced nephrotoxicity compared with that of other 5-HT_3_RAs in the present retrospective clinical study and pharmacovigilance analysis. To the best of our knowledge, this is the first study to report the effect of palonosetron on CDDP-induced nephrotoxicity in patients receiving CDDP, 5-FU, and triple antiemetic therapy.

In the present study, the fold change in Scr and BUN following CDDP administration in patients treated with palonosetron was significantly lower than that in patients treated with ramosetron (Fig. 1). In addition, the incidence of nephrotoxicity (grade ≥ 1) in patients receiving palonosetron was significantly lower than that in patients treated with ramosetron (Table 2), and grade ≥ 3 nephrotoxicity was observed in one patient treated with ramosetron. Moreover, the effect of palonosetron on CDDP-induced nephrotoxicity was validated in patients with the triple antiemetic therapy using the JADER database. We could not get similar results in comparison between palonosetron and ramosetron because there were not enough cases of ramosetron use in JADER database to fully evaluate the effect of ramosetron (Table 3). These results are suggestive of better potential of palonosetron than ramosetron against CDDP-induced nephrotoxicity in patients with triple antiemetic therapy.

CDDP is excreted by the kidneys, and specifically accumulates in the renal proximal tubules [11]. CDDP is mainly transported to renal tissues via organic cation transporter 2 (OCT2) at the renal basolateral membrane [12, 13], whereas CDDP is excreted into urine through multidrug and toxin extrusion protein transporter 1 (MATE1), which is localized on the apical membrane [14], indicating that OCT2 and MATE1 should be responsible for CDDP-induced nephrotoxicity. As shown in a previous study using the mice model of CDDP-induced nephrotoxicity [8], the concomitant use of a first-generation 5-HT_3_RA (ondansetron, granisetron, or ramosetron) significantly increased CDDP accumulation in the kidneys and worsened renal damage. Conversely, the concomitant use of palonosetron had no effect on renal function compared with the use of CDDP alone. An uptake study in hMATE1-expressing HEK293 cells revealed that the first-generation 5-HT_3_RAs have a lower IC_50_ than palonosetron, thus, palonosetron is thought to have weaker MATE1 inhibitory activity than the first-generation 5-HT_3_RAs [8]. Furthermore, palonosetron was reported to interfere with OCT2 activity [15]. Taking these findings into consideration, we speculate that palonosetron ameliorated CDDP-induced nephrotoxicity by decreasing the accumulation of CDDP in the kidney via OCT2. However, further studies are needed to elucidate the detailed mechanism of protective effect of palonosetron against CDDP-induced nephrotoxicity.

Nevertheless, inhibition of OCT2-mediated transport of CDDP by 5-HT_3_RAs is expected to increase its plasma concentration, which may lead to hematological side effects associated with CDDP. In the present study, there was no significant difference in the incidence of hematological toxicity between patients treated with palonosetron and ramosetron (Table 2). However, a previous study reported that combination treatment with palonosetron did not affect the blood levels of CDDP in mice [8]. Thus, it is likely that plasma concentration of CDDP is not affected by co-administration of palonosetron and/or ramosetron.

CINV results in significant morbidity, adversely affects patient’s quality of life, and leads to poor compliance with treatment regimens [16–18]. The present study showed that the incidence rates of CINV in patients treated with palonosetron were significantly lower than in those receiving ramosetron during both delayed phase and overall phase (Fig. 2). Palonosetron has a longer half-life in plasma and a higher binding affinity than first-generation 5-HT_3_RAs [19]. A previous study reported that palonosetron was significantly more effective than tropisetron (a first-generation 5-HT_3_RA) in controlling delayed emesis in patients receiving high dose of CDDP [20]. Moreover, palonosetron has been reported to be cost-effective treatment strategy for the prophylaxis of CINV in highly and moderately emetogenic chemotherapy compared to other 5-HT_3_RAs [21, 22]. Therefore, these findings suggested palonosetron as a potential alternative for controlling CINV in patients receiving highly emetogenic chemotherapy, including CDDP regimen.

This study had several limitations. First, it remains unclear whether palonosetron directly inhibits OCT2-mediated renal uptake of CDDP. Second, the plasma concentration of CDDP was not assessed in the present study. Finally, the level of evidence was poor because this was a retrospective study that included a small number of patients from a single institution. Thus, additional in vitro and in vivo studies with large and diverse samples are warranted to validate our findings and to reveal altered pharmacokinetics of CDDP by co-administration of palonosetron.

## Conclusions

In conclusion, our study is the first to demonstrate that palonosetron is more effective in preventing CDDP-induced nephrotoxicity and CINV than other 5-HT_3_RAs. The present findings provide important information to optimize the current treatment regimens to minimize CDDP-induced nephrotoxicity.

## Data Availability

All data generated or analyzed during this study are included in this published article.

## Abbreviations

5-FU: fluorouracil
5-HT_3_RA: 5-hydroxytryptamine type 3 receptor antagonist
ALT: alanine aminotransferase
ANC: absolute neutrophil count
AST: aspartate aminotransferase
BUN: blood urea nitrogen
CDDP: cisplatin
CI: confidence interval
CINV: chemotherapy-induced nausea and vomiting
CTCAE: Common Terminology Criteria for Adverse Events
Hb: hemoglobin
JADER: Japanese Adverse Drug Event Report
MATE1: multidrug and toxin extrusion protein transporter 1
MedDRA: Medical Dictionary for Regulatory Activities
NSAIDs: non-steroidal anti-inflammatory drugs
OCT2: organic cation transporter 2
PLT: platelet
PPI: proton pump inhibitor
ROR: reporting odds ratio
Scr: serum creatinine
WBC: white blood cell
